# Pathological and Tissue-Based Molecular Investigation of Granulomas in Cichlids Reared as Ornamental Fish

**DOI:** 10.3390/ani12111366

**Published:** 2022-05-26

**Authors:** Luciana Mandrioli, Victorio Codotto, Giulia D’Annunzio, Enrico Volpe, Francesca Errani, Yoshinobu Eishi, Keisuke Uchida, Maria Morini, Giuseppe Sarli, Sara Ciulli

**Affiliations:** 1 Department of Veterinary Medical Sciences, Alma Mater Studiorum University of Bologna, 40126 Bologna, Italy; victoriocodotto@gmail.com (V.C.); giulia.dannunzio2@unibo.it (G.D.); enrico.volpe2@unibo.it (E.V.); francesca.errani2@unibo.it (F.E.); maria.morini@unibo.it (M.M.); giuseppe.sarli@unibo.it (G.S.); sara.ciulli@unibo.it (S.C.); 2 Department of Human Pathology, Graduate School and Faculty of Medicine, Tokyo Medical and Dental University, Yushima 1-5-45, Tokyo 113-8519, Japan; eishi.path@tmd.ac.jp (Y.E.); uchida.path@tmd.ac.jp (K.U.)

**Keywords:** *Cutibacterium acnes*, debilitating disease, granuloma, immunohistochemistry, Mycobacteria, ornamental fish, *Propionibacterium acnes*

## Abstract

**Simple Summary:**

The global ornamental fish trade has an estimated value of USD 15–30 billion per year and more than a 10% average annual growth. Despite their economic importance, the management of ornamental fish is challenged by a paucity of information, including data on the fish health status. Pathological and microbiological investigations were conducted on ornamental cichlids sampled during routine management activities held at an aquarium commercial facility, in order to evaluate the presence of granuloma in the organs. *Cutibacterium acnes* and *Mycobacterium* spp. were detected by molecular methods and immunohistochemistry. These bacteria represent potential zoonotic agents, and the advancement of their knowledge could significantly improve the management of ornamental fish and reduce the risk of exposure for people, such as hobbyists, fish handlers, aquarists, and dedicated personnel.

**Abstract:**

Cichlids include hundreds of species with a high economic value for aquaculture. These fish are subjected to intensive trade and farming that expose them to the risk of infectious diseases. This work focuses on ornamental cichlids held in an aquarium commercial facility presenting emaciation, in order to evaluate the presence of lesions in fish skin and organs. The fish were sampled during routine management activities and subjected to pathological and molecular investigations. The presence of lymphocystis disease virus, typically associated with cutaneous nodular disease, was ruled out. Histologically, they presented granulomas in the spleen, sometimes extending to the other visceral organs. Bacterial heat-shock protein 65 PCR products were detected in tissues associated, in the majority of cases, with granulomas; molecular investigation identified *Mycobacterium* spp. in two cases and *Cutibacterium acnes* in seven cases. Immunoreactivity to anti-Mycobacterium and anti-C. acnes antibodies was detected within granulomas. The presence of *C. acnes* within granuloma is elucidated for the first time in fish; however, similarly to what is found in humans, this bacterium could be harmless in normal conditions, whereas other contributing factors would be required to trigger a granulomatogenous response. Further confirmation by bacterial culture, as well as using large-scale studies in more controlled situations, is needed.

## 1. Introduction

African cichlids are highly appreciated as “colorful rock dwelling fishes” by aquarists worldwide. The ornamental fish export trade is mostly based on fish from the Lake Malawi, with an average of 28,000 live fish being exported annually, valued at USD 218,000 in 2020 [1]. These fish are subjected to intensive trade and farming, which greatly expose them to the risk of the emergence of infectious diseases. However, despite their economic importance, the management of ornamental fish is challenged by a paucity of information on the causes of subtle diseases [2]. These disorders often present as debilitating diseases, whose etiology has been poorly investigated. Fish can display nonspecific signs of disease, such as emaciation, exophthalmia, keratitis, and skin lesions, as well as nodules and black spots. Mycobacterial infection is probably the most common chronic disease affecting aquarium fish species [3]. Cichlids are known for territorial behavior and sexual aggression, making these animals prone to skin damage, which might provide a portal for bacterial penetration and the subsequent transformation of dermal mycobacteriosis into systemic granulomatous infection [4]. Regarding viral diseases, those associated with viruses in the Iridoviridae family are reported in ornamental fish. Among viruses of this family, Megalocytivirus and Ranavirus are considered highly pathogenic iridoviruses and frequently associated with highly mortality outbreaks, whereas Lymphocystivirus is mainly associated with a self-limiting cutaneous nodular disease [5,6]. Thanks to high species diversity and a broad range of speciation mechanisms, cichlid fish represent a textbook model in parasite evolutionary biology. Despite their importance, among the biological agents of disease, cichlid parasites remain understudied [7].

Moreover, the relevance of ornamental cichlids is not counterbalanced by literature references regarding pathologic conditions, with only a limited amount of papers being available [8,9,10,11,12,13,14].

This study focuses on fish belonging to six ornamental cichlid species and the genus *Placidochromis* sp., which were sampled during routine fish management activities. Our goal is to increase knowledge about debilitating diseases in African cichlids reared as ornamental fish. To achieve this, a different approach was performed to investigate the molecular and pathological characteristics and search for the most probable agents of disease.

## 2. Materials and Methods

### 2.1. Animals Clinical History and Sampling

This investigation focuses on African ornamental cichlid belonging to the following six species: *Aulonocara jacobfreibergi*, *Maylandia estherae*, *Mylochromis guentheri*, *Maylandia pulpican*, *Maylandia lanisticola*, and *Petrotilapia xanthos*, as well as the genus *Placidochromis* sp.

The fish were kept in 250 L tanks, equipped with independent filters and holed bricks used for shelter by the animals, natural photoperiod, and heated well water; they were fed a commercial diet (Skretting©, Stavanger, Norway). The water’s physical–chemical parameters were pH 7.7 ± 0.3, water hardness 280 ppm, NH_4_ < 0.05 ppm, and NO_2_ < 0.01 ppm (Hanna instruments, Woonsocket, RI, USA).

In 2019, during the routine management activities, some fish presented black spots and cutaneous nodules, whereas others showed debilitated status, emaciation, and poor growth. An investigation regarding the possible underlying diseases was conducted. Two fish samplings were conducted in August and October. During the first sampling, the water temperature was 27–28 °C. The second sampling had a consistent temperature of 24 °C. All fish in the first sampling came from breeding activities within the company, whereas the second sampling included a subject that was purchased from another fish farmer and used for breeding. The fish were sampled with a purposive sampling. The selection of the subjects was based on visual and behavioral abnormalities: twelve debilitated subjects, which were emaciated and inclined to be isolated from the group, with some of them having nodules and/or dark spots on their skin, were collected. The fish were individually packaged and transported alive in an insulated container to the laboratory for analyses. Then, they were euthanized one at a time with a lethal dose of anesthetic 2-phenoxyethanol (200 ppm), diluted in the bag used for transport, weighed (analytical balance, Scaltec, Heiligenstadt, Germany), and measured. The weight of the twelve fish ranged from 3.1 g to 13.6 g, with the total length varying from 8.5 cm to 10 cm.

Afterwards, sampling was performed with sterile instruments (forceps and scissors) for molecular investigations. Spleen, liver, kidney, heart, intestine, and any pathological tissue, such as cutaneous nodules, were collected.

### 2.2. Histology and Histochemistry

Sampled organs of all 12 fish were fixed in 10% (*v*/*v*) phosphate-buffered formalin (Titolchimica, Rovigo, Italy), according to standard procedures, paraffin embedded (embedding center, Histoline, Milan, Italy), and then cut into 3 μm sections (rotative microtome, Leica Microsistem, Milan, Italy). Then, the sections were stained with Hematoxylin–Eosin (H&E, Histoline, Milan, Italy). Concurrently, sections of the spleen were stained with Gram (Gram Kit, Histoline Laboratories, Milan, Italy) and, for the detection of acid-fast bacteria, Ziehl–Neelsen kit (Histoline Laboratories, Milan, Italy). Archived positive controls for Gram and acid-fast staining were included.

### 2.3. Molecular Investigation for Lymphocystis Disease Virus (LCDV)

Subjects with nodules or skin alterations (*n* = 9) were analyzed for the lymphocystis disease virus (LCDV) by molecular method. The skin tissue, or a portion of the sampled fin, was subjected to DNA extraction, using approximately 20 mg of tissue. DNA was extracted using the Purelink genomic DNA extraction kit (Invitrogen, Carlsbad, CA, USA), according to the manufacturer’s instructions. The LCDV investigation was then conducted on these samples using a nested PCR assay. The first amplification step was performed using the LF7/LC1R primers [15,16], and the second amplification step used the LCDV qPCR F1 and LCDV qPCR R3 primers [17]. For each amplification, a reaction mix containing 1× PCR Buffer, 1 mM MgCl_2_, 200 µM dNTP, 0.4 µM of each primer, 1.25 U of Taq polymerase, and 5 µL of DNA in a final volume of 25 µL was performed. The first amplification step was conducted at 94 °C for 5 min, 45 cycles consisting of denaturation at 94 °C for 2 min, annealing at 55 °C for 2 min, extension at 72 °C for 2 min, and a final extension at 72 °C for 10 min. The second amplification step was conducted at 95 °C for 5 min, 45 cycles consisting of denaturation at 95 °C for 15 s, annealing at 50 °C for 30 s, extension at 72 °C for 30 s, and a final extension at 72 °C for 10 min. Positive and negative controls were run together with the samples. The positive control consists of DNA extracted from pathological tissue collected from fish previously confirmed as true positive [17]. Nested PCR products were visualized by electrophoresis on 1.5% agarose gel by running the samples together with a reference molecular marker (100 bp ladder, Invitrogen, Carlsbad, CA, USA).

### 2.4. Molecular Investigation for Bacteria

Due to the finding of granulomas, as revealed by the histopathological investigation, the presence of *Mycobacterium* sp. was hypothesized; then, mycobacterial DNA presence was investigated in frozen samples (pool made of liver, spleen, heart, and kidney) for three cases and formaldehyde-fixed paraffin-embedded (FFPE) samples for nine cases. Frozen samples (cases 10, 11, and 12) were processed for DNA extraction using the Purelink Genomic DNA kit (Invitrogen, Carlsbad, CA, USA), following the manufacturer’s instructions. FFPE samples (cases 1 to 9) were processed for DNA extraction using the Purelink Genomic DNA kit (Invitrogen, Carlsbad, CA, USA), following the manufacturer’s instructions, with minor modifications. Particularly, unstained spleen sections, serial to sections showing granulomas when histologically present (cases 1, 2, 5, 6, and 9), were used for DNA extraction. In two cases for which the spleen was not valuable, perivisceral adipose tissue sections (cases 3 and 4) were used. Five to ten mg of sliced FFPE tissue was placed in 1 mL of xylene (J.T. Baker, Phillipsburg, NJ, USA), and a pre-extraction step to remove paraffin from the sample was applied, as previously described [18]. Samples were deparaffinized in xylene for 5 min; following centrifugation, samples were washed twice in 100% ethanol. The pellet was dried at 37 °C for 10 min, and DNA extraction was subsequently undertaken, using the aforementioned kit. Bacterial presence was investigated with a PCR targeting the HSP65 gene using primers common to all mycobacteria [19]. For the amplification, a reaction mixture was assembled containing 1× PCR buffer, 1 mM MgCl_2_, 200 µM dNTP, 0.4 µM of each primer, 2.5 U of Taq polymerase, and 5 µL of DNA in a final volume of 25 µL. The amplification cycle was conducted at 94 °C for one minute, 45 cycles consisting of denaturation at 94 °C for one minute, annealing at 60 °C for one minute, extension at 72 °C for one minute, and a final extension step at 72 °C for 10 min. The PCR results were visualized by electrophoresis on 1.5% agarose gel by running the samples with a reference marker (100 bp ladder Invitrogen). PCR products of positive samples were purified using the ExoSAP-IT PCR product cleanup reagent (Invitrogen, Carlsbad, CA, USA) and sequenced through the Bio-Fab Sequencing Service (Rome, Italy). The sequences were then manually corrected and subjected to BLAST analysis (http://blast.ncbi.nlm.nih.gov/Blast.cgi accessed on 18 January 2022) for identification.

### 2.5. Immunohistochemistry (IHC)

A polyclonal antibody against *Mycobacterium bovis* (Bacillus Calmette-Guerìn, BCG, code no. B 0124 Dako, Glostrup, Denmark), previously applied to detect mycobacteria in fish granulomas [20,21], was employed. Endogenous peroxidase inhibition was made with 3% H_2_O_2_ methanol solution. The antigen retrieval was made by using a microwave oven at 750 W, 2 cycles × 5 min. Preincubation with a blocking solution (10% normal goat serum and phosphate-buffered saline solution) was performed for 30 min and incubated overnight at 4 °C, with a primary antibody diluted at 1:3000 in a blocking solution. The secondary anti-rabbit antibody was incubated at 1:200 dilution for 30 min, followed by a revelation system with an avidin–biotin complex (ABC) kit developed with diaminobenzidine (DAB) chromogen for 90 s and counterstained with Papanicolaou hematoxylin.

To study *C. acnes* (formerly *Propionibacterium acnes*) [22], polarized slides were submitted to IHC with a previously validated *C. acnes*-specific monoclonal (PAB) antibody that reacts with lipoteichoic acid, the cell membrane constituent of the bacterium, in formalin-fixed and paraffin-embedded (FFPE) tissues. The diagnostic accuracy, in terms of specificity and sensibility of PAB, was fully demonstrated by Negi et al. [23]. This monoclonal antibody reacted with all strains of *C. acnes*, and there was no cross-reactivity with other bacteria [23]. IHC with a commercially available PAB antibody (#D371-3, MBL, Nagoya, Japan) was performed by Leica BOND-III (Leica Microsystems Inc., Tokyo, Japan) using a BOND polymer refine detection kit (#DS9800, Leica Microsystems Inc.), as described in the study by Isshiki and colleagues [24]. Deparaffinization, peroxidase inhibition, antigen retrieval with BOND Epitope Retrieval Solution 1 (#AR9961, Leica Microsystems Inc.) at 100 °C for 60 min, incubation with PAB antibody (diluted 1:500) at room temperature for 8 min, and counterstaining with Mayer’s hematoxylin were performed according to the manufacturer’s instructions.

Internal positive and negative control slides were processed in parallel by replacing the primary antibody with a non-reactive isotype-matched antibody. Positive controls consist of fish and human tissues that were previously confirmed as positive to anti-Mycobacterium and PAB antibodies, respectively [21,23].

## 3. Results

### 3.1. Gross and Microscopical Findings

**Figure 1:**
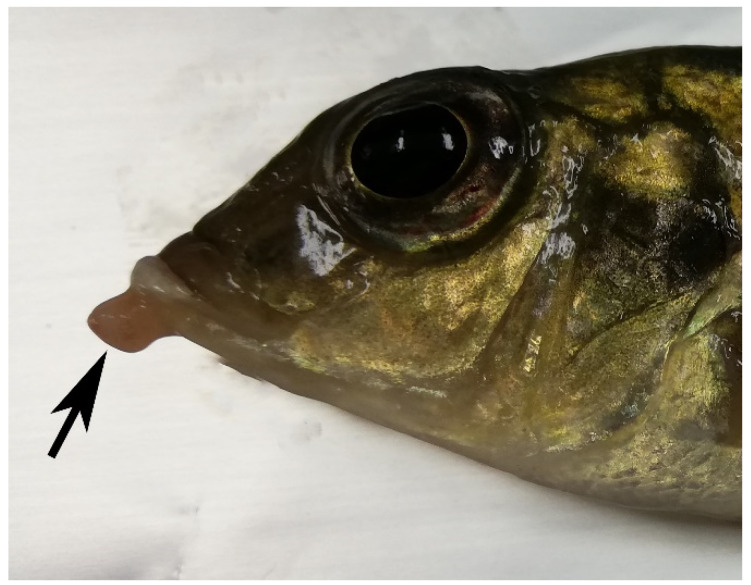
Case 7. Cichlid fish. *M. guentheri*. Region of the mouth, showing a pinkish nodule bulging from the oral mucosa (black arrow).

Overall, the fish were emaciated, thin, and had scarce adipose tissue. The information regarding the sex, size, and weight, as well as gross and microscopic lesions detected, are summarized in Table 1. Five fish, including the species *A. jacobfreibergi*, *M. estherae*, *M. pulpican*, and genus *Placidochromis* sp., were revealed to have round or irregularly-round black areas on their integument and fins, ranging from 2 to 5 mm in size. In addition, three fish, including the species *M. estherae*, *M. guentheri*, and *P. xanthos*, showed white or pinkish nodules on the skin, fins, and/or oral mucosa (Figure 1). In one fish of *M. pulpican* (case 9), white-yellowish multifocal granulomas were observed in the spleen and perivisceral adipose tissue.

On the basis of the gross findings, in combination with the histological examination referring to the gonads, the sex was detected in seven out of twelve fish (Table 1).

**Figure 2:**
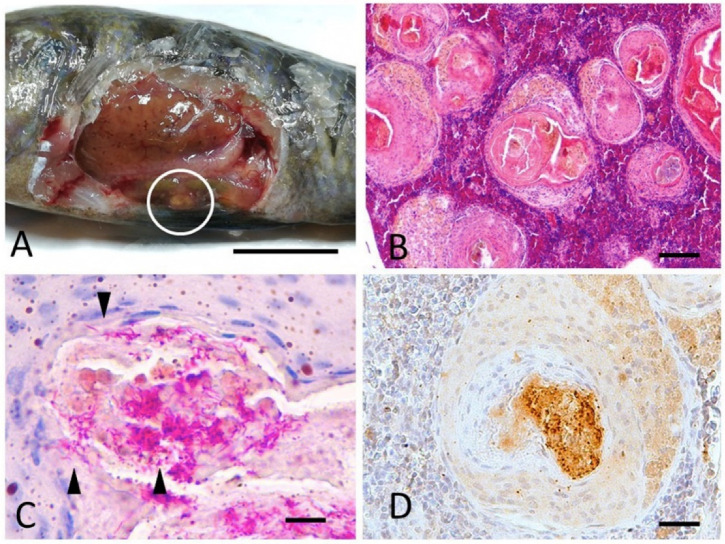
Case 9. Cichlid fish. *M. pulpican*. (**A**) Viscera. White-yellowish multifocal granulomas in the perivisceral adipose tissue and visceral organs (white circle), bar 1 cm. (**B**) Spleen. Multifocal, coalescent granulomas developing from melano-macrophagic centers (MMC) dispersed within the splenic parenchyma. Hematoxylin–Eosin staining, bar 300 µm. (**C**) Spleen, acid-fast bacilli (arrowheads) are present within granulomas. Ziehl–Neelsen stain, bar 30 µm. (**D**) Spleen, granular labelling to anti-Mycobacterium antibody within granuloma. Immunohistochemistry, DAB staining, bar 100 µm.

**Figure 3:**
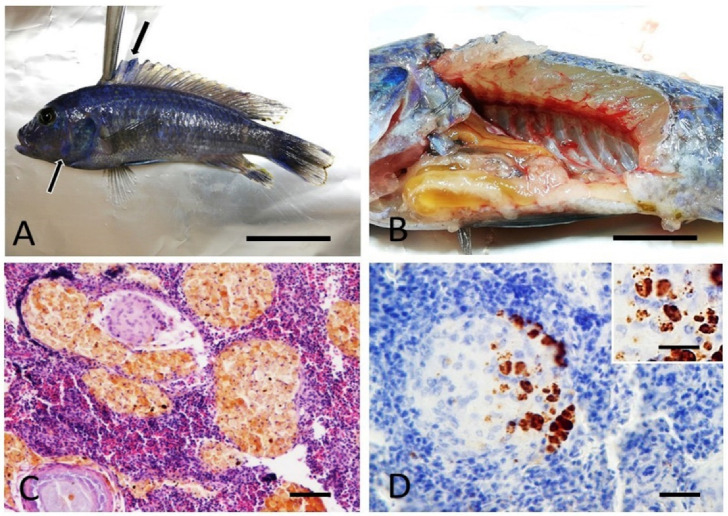
Case 2. Cichlid fish. *A. jacobfreibergi*. (**A**) Skin. Multifocal, irregularly round black areas (black arrows) of the integument and fins, bar 2 cm. (**B**) Viscera. Scarce perivisceral adipose tissue and intestinal viscera showing a serous content, bar 1 cm. (**C**) Spleen. Multifocal, coalescent granulomas dispersed through the splenic parenchyma. Hematoxylin–Eosin staining, bar 300 µm. (**D**) Spleen, variably-sized round so-called “Hamazaki-Wesenberg” bodies immunolabeled with anti-PAB antibody within macrophages forming the granulomas. Immunohistochemistry, DAB staining, bar 100 µm. Inset shows higher magnification of anti-PAB immunoreactivity. Immunohistochemistry, DAB staining, bar 30 µm.

**Figure 4:**
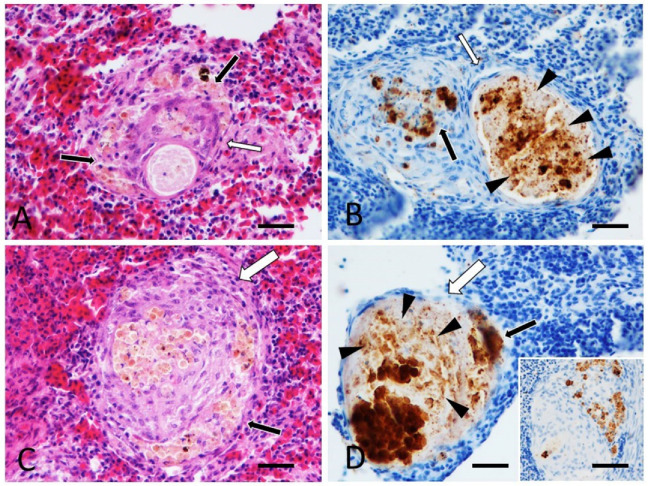
Cases 1 (**A**,**B**) (*A.* *jacobfreibergi*) and 5 (**C**,**D**) (*M. estherae*). Spleen. Comparison between H&E ((**A**,**C**) bar 100 µm) and IHC ((**B**,**D**) bar 100 µm) of MMC and granulomas labelling PAB antibody. (**A**,**C**) Development of granulomas within MMC. (**B**,**D**) IHC signal associated with macrophages of MMC (black arrows) and granular and specific immunoreactivity (arrowheads) within granulomas (white arrows) arising inside the MMC. Inset: negative control. MMC at upper right shows an aspecific signal, the granuloma in the center does not show staining (inset: bar 300 µm).

At histology, only the spleen was consistently valuable for the majority of cases (nine out of 12 cases), due to the small size of the fish and small quantity of most of the internal organs (i.e., kidney). Microscopically, the spleen of six fish of the species *A. jacobfreibergi*, *M. estherae*, *M. pulpican*, and *M. lanisticola* (cases 1, 2, 5, 6, 9, and 11) showed multifocal, coalescent epithelioid granulomas, with occasional central necrosis and concurrent hyperplasia of melanomacrophagic centers (MMC) (Figure 2B and Figure 3C). The development of granulomas was frequently observed within MMC (Figure 3C and Figure 4A,C). In case 9, the spleen showed acid-fast bacteria within granulomas (Figure 2). In other cases, including 1, 2, 5, 6, and 11, the granulomas were Ziehl–Neelsen negative. All granulomas contained Gram-positive granular material consistent with bacteria. The cutaneous nodules were histologically consistent with the presence of mature granulation tissue and concurrent mild, chronic, and lymphocytic dermatitis; signs of hypertrophy of fibroblasts, suggestive of an LCDV infection, were absent. The black areas of the skin matched with focal hypermelanosis and were not associated with intralesional parasites.

### 3.2. Molecular Analyses

Concerning the molecular detection of LCDV, the tested skin samples of nine subjects were negative.

Regarding bacteria identification, nine out of the 12 samples tested positive for the PCR targeting the HSP65 gene of *Mycobacterium* sp. However, sequencing of the PCR products showed that only some of them were ascribable to the *Mycobacterium* genus. BLAST analysis of the obtained sequences identified mycobacteria in two samples (cases 9 and 11), including species *M. pulpican* and *M. lanisticola*, as well as *C. acnes* in the other seven samples (cases 1, 2, 3, 4, 5, 6, and 8), including the species *A. jacobfreibergi*, *M. estherae*, and *M. guentheri*, as well as the genus *Placidochromis*. In particular, the sequences obtained from the samples of cases 1, 2, 3, 4, 5, 6, and 8 showed ≥98.5% nucleotide identity with *P. acnes* ATCC 11828 (GenBank accession number CP003084); most of these cases showed splenic granulomas; however, in at least one case (case 8), it was not possible to detect granulomas in the splenic tissue subjected to histological analysis. Sequences from the samples of cases 9 and 11 showed the highest similarity with *M. chelonae* (100% nucleotide identity with JX154110) and *M. parascrofulaceum* (98.4% nucleotide identity with AY337276), respectively.

### 3.3. Immunohistochemistry (IHC)

A granular immunoreactivity to the anti-Mycobacterium antibody was detected within granulomas in case 9, referring to *M. pulpican* (Figure 2D).

In the spleens of cases 1, 2, 5, 6, and 11 (*A. jacobfreibergi*, *M. estherae*, and *M. lanisticola*), variable-sized, round, so-called “Hamazaki-Wesenberg” bodies were immunolabeled with PAB antibody within macrophages forming the granulomas (case 2, Figure 3D). In particular, all observed granulomas immunoreacted, and, in most of them, immunoreactivity was marginal and non-associated with the necrotic centers. In some cases (1, 5, and 6), an intense IHC signal was detected in the macrophages forming the MMC, presumably associated with constitutive cell pigments (Figure 4B,D). However, within granulomas, there was also a more granular and clearly specific labeling, as shown by the comparison with the negative control (Figure 4B,D).

## 4. Discussion

Cichlid fishes (Cichlidae) are one of the most worldwide species-rich and widespread families of vertebrates, representing a substantial part of the ornamental fish trade and industry [1]. Despite that, a paucity of information on the diseases frequently encountered in rearing is available.

Ornamental fish can display nonspecific signs of disease, such as emaciation, exophthalmia, keratitis, and skin lesions, which may present as nodules and black spots, that are associated with different causes.

Regarding viral diseases, in ornamental fish, including cichlids, several infections, due to iridovirus-like microorganisms, are reported [25]. Particularly, lymphocystis outbreaks are characterized by a low mortality rate; however, the obvious cutaneous lesions make the subjects not suitable for sale [3]. The lymphocystis disease virus generally infects dermal fibroblasts, causing their hypertrophy; the internal organs or gills are rarely affected [26]. LCDV commonly presents with white pigmented masses, grossly visible mainly on the pectoral and dorsal fins [27], similar to those observed in some of the fish investigated in this study; however, in our cases, LCDV was not detected. Histologically, the cutaneous nodules were consistent with chronic dermatitis, which matched the negative molecular results. As these fish species are extremely territorial and frequently fight amongst each other, the observed chronic dermatitis could be related to repeated post-traumatic events.

Several bacteria that may cause damage to the fish are found naturally in their microbiota or environment, and they are usually in balance with their hosts. In cases of worsening environmental conditions, skin injuries, and/or host immune system impairment, these bacteria may become pathogenic. Mycobacterial infections are the most common chronic disease affecting ornamental species, and they are reported in more than 150 species [3,28]. These infections are zoonotic and, in humans, may present as cutaneous ulcers that struggle to heal [3]. As a matter of fact, fish mycobacteriosis is sustained by three species most of the time: *M. marinum, M. fortuitum,* and *M. chelonae* [28]. Other, less frequently isolated species are *M. abscessus, M. gordonae, M. conceptionense, M. parascrofulaceum*, and *M. senegalense* [29]. In this study, the results aligned with those reported, with both *M. chelonae* and *M. parascrofulaceum* detected in our fish subjects. A previous study investigated the presence of mycobacteria in freshwater ornamental fish, including six species of Cichlids, with signs of chronic disease, such as persistent cutaneous lesions, abdominal swelling, and overall poor general health. This study pointed out the presence of a granulomatous inflammation associated with acid-fast bacteria in 41% of them. However, the characterization of bacteria associated with granulomas was not conducted [30].

Nevertheless, granulomas can be caused by a variety of bacteria, besides mycobacteria, such as *Francisella* spp., *Nocardia* spp., *Piscirickettsia salmonis*, and atypical isolates of *Aeromonas salmonicida*. [31,32,33]. Particularly, a Francisella-like bacterium infection has been described in association with visceral granulomas in ornamental cichlid [34].

In our study, Mycobacterium infections were found in only two out of six subjects with granulomas. In seven cases, of whom, four showed granulomas, *C. acnes* was identified from FFPE and/or frozen samples through molecular analysis and immunohistochemistry. This finding was interpreted as an intralesional presence, as it was found within the visceral organs of fish placed in sterile conditions for tissue sampling. Recently, this bacterium has been found within fish and the aquatic environment [35,36].

*C. acnes* is an anaerobic, commensal, lipophilic, Gram-positive bacterium. *C. acnes*, classically studied as a human bacterial agent of acne vulgaris, is an opportunistic pathogen, with a likely underestimated role in the development of disease. In addition to acne, it is associated with other human diseases, including prosthetic joint infections, prostate cancer, intervertebral disks surgery, and sarcoidosis [37,38]. It is now the second-most frequent pathogen, after coagulase-negative staphylococci, isolated from infected internal cerebral ventricular bypasses, and rates of infection with this bacterium have increased from 1.5% to 38%. *C. acnes* has also been found in blood cultures, where it may represent up to 80% of the isolated anaerobes [38]. However, its role in human diseases is still debated, and its wide colonization of human organs suggests that the bacterium does not harm the human host, at least, not under normal circumstances [22].

Analogously, *C. acnes* was recently detected through NGS analysis in the internal organs of marine fish without external or internal pathological changes and could suggest its harmless nature; however, three well-known bacterial pathogens, i.e., *Photobacterium damselae, Vibrio harveyi*, and *Streptococcus iniae*, were also found in these organs [36]. Despite the previous studies stating that healthy fish internal organs should be sterile, lately, studies have reported that bacteria are being found in healthy kidneys and livers [36,39]. However, assuming the organs were macroscopically healthy, a histopathological investigation was not performed in these studies; so, the presence of potential tissue reactions as granuloma in association with the bacterial presence was not possible to exclude.

To demonstrate how *C. acnes* causes infection or colonization, suggestions have been made to use a combination of techniques, including immunohistochemistry [40]. In our study, complementary techniques, including PCR, histochemical stainings, and IHC, were performed to detect bacterial components. According to the experience of these authors [21], the sampling of tissues using combined methods, such as IHC and molecular techniques, is highly recommended to maximize the results. Gram-positive bacterial aggregates, which were concurrently immunoreactive to the anti-PAB antibody and identified by molecular analysis, were detected within granulomas. As a whole, these findings suggest that *C. acnes* could be involved in eliciting a granulomatous reaction. However, we also found *C. acnes* in a case without granulomas’ evidence; this could suggest that, similarly to what has been found in humans, this bacterium could be harmless in normal conditions, and other contributing factors would be required to trigger a granulomatogenous response.

The immunohistochemical results obtained in our cases resemble the so-called “Hamazaki-Wesenberg” bodies described in human lymph nodes from patients affected by granulomatous conditions. In human tissues, both immunohistochemistry and electron microscopy findings suggest that these bodies are intracellular bacteria [23].

Sarcoidosis is an enigmatic, multi-systemic human disease of unknown origin: insights into the etiology and pathogenesis have been elusive [41]. It is postulated to be a multifactorial disease caused by chronic antigenic stimulation [42]. Genetic background may have a predisposing role; moreover, microbial infections, specifically *C. acnes* and *Mycobacteria* spp., pine pollen, and air pollutants, are increasingly regarded as strong environmental trigger candidates [43]. Sarcoidosis is characterized by the development and accumulation of epithelioid, non-caseating granulomas typically found in the lungs; however, sarcoid granulomas can be present almost anywhere in the body [43].

Considering animal models of sarcoidosis, zebrafish (*Danio rerio*) have been employed to study its pathogenesis. Nevertheless, there is no universally accepted animal model for human sarcoidosis, largely because animals, other than horses, do not develop spontaneous sarcoidosis, and the link between human gene polymorphisms and disease prevalence has not been established with the aim to be recapitulated in the animal genetic manipulation [44].

Regarding the use of the term sarcoidosis in fish, it was used in a recent paper by He and colleagues [33] to describe multiple granulomas that were dispersed through several organs in a species of commercial interest, the largemouth bass (*Micropterus salmoides*), as well as the association with the bacterium *Nocardia seriolae*, a well-known agent able to induce granulomatous reaction in fish.

By using the term sarcoidosis, He and colleagues [33] placed emphasis on the relationship among the granuloma and bacteria, other than mycobacteria, in an attempt to identify and recognize this condition.

## 5. Conclusions

In conclusion, the detection of bacteria that are able to elicit granulomatous reaction represents a significant, but still debatable, finding in this study. We advise adding *C. acnes*, as well as the yet widely-known *Mycobacterium* spp., to the list of bacteria that are associated with granulomatous reaction. The well-known zoonotic role of the mycobacteria found in aquarium fish still raises concerns regarding proper management by fish handlers, aquarists, and dedicated personnel.

Due to the zoonotic disease potential and impossibility of elimination of mycobacteria from the aquatic environment, depopulation and aquaria disinfection are recommended, as they are the only current measures for containing disease outbreaks.

The results of this study suggest, other than the yet known Mycobacteria spp., the presence of *C. acnes* within fish visceral granulomas, incidentally found during routine health surveillance activity in an ornamental fish company. In the present study, we successfully applied the PAB antibody, in order to detect *C. acnes* in formalin-fixed paraffin-embedded tissue sections in cichlids.

However, the role of the newly detected *C. acnes* needs to be investigated in depth, using large-scale studies, with combined laboratory techniques, including the isolation of live bacteria in natural outbreaks and under more controlled experimental conditions. In addition, Koch’s postulate must be tested to establish causality. Indeed, further research is necessary to understand the pathogenetic mechanisms and yet unknown, highly probable multifactorial etiology that underlie the granulomatous condition that also affects ornamental fish.

## Figures and Tables

**Table 1:** Synoptic table of ornamental fish species investigated in this study.

Case Number	Genus/Species	Weight (g)	Length (cm)	Sex	Gross Findings			Histological Findings	Molecular Pathogen Detection	
					Black Spots	Cutaneous Nodules	Visceral Granulomas	Splenic Granulomas	LCDV	Bacterium Identified
1	*A.* *jacobfreibergi*	7.90	10	F	present	absent	absent	present	Negative	*C. acnes*
2	*A. jacobfreibergi*	3.10	9.5	F	present	absent	absent	present	nd	*C. acnes*
3	*Placidochromis* sp.	6.40	9	F	present	absent	absent	nv	nd	*C. acnes*
4	*Placidochromis* sp.	7.75	8.5	F	absent	absent	absent	nv	nd	*C. acnes*
5	*M. estherae*	10.38	9.5	nv	present	present	absent	present	Negative	*C. acnes*
6	*M. estherae*	11.64	9.5	nv.	absent	absent	absent	present	Negative	*C. acnes*
7	*M. guentheri*	11.04	9.5	F	absent	present	absent	absent	Negative	Negative
8	*M. guentheri*	5.30	8.5	nv	absent	absent	absent	absent	Negative	*C. acnes*
9	*M. pulpican*	8.61	9	F	absent	absent	present	present	Negative	*M. chelonae*
10	*M. pulpican*	10.29	8.5	M	absent	absent	absent	nv	Negative	Negative
11	*M. lanisticola*	9.27	9	nv	present	absent	absent	present	Negative	*M. parascrofulaceum*
12	*P. xanthos*	13.60	10	nv	absent	present	absent	absent	Negative	Negative

Abbreviations: nv: not valuable; nd: not determined; F: female; M: male.

## Data Availability

The data presented in this study are available from the corresponding author upon reasonable request.
